# Reversal of diastolic flow in epididymo-orchitis: a red flag for impending testicular infarction

**DOI:** 10.1093/bjrcr/uaag028

**Published:** 2026-07-21

**Authors:** Yilin Liu, Arpit Talwar

**Affiliations:** Faculty of Medicine, Dentistry & Health Sciences, The University of Melbourne, Melbourne, Victoria 3010, Australia; Medical Imaging Department, St Vincent’s Hospital Melbourne, Melbourne, Victoria 3065, Australia; Medical Imaging Department, St Vincent’s Hospital Melbourne, Melbourne, Victoria 3065, Australia

**Keywords:** epididymo-orchitis, testicular infarction, reversal of diastolic flow, spectral Doppler ultrasound, acute scrotum

## Abstract

Epididymo-orchitis usually responds to antibiotics, yet a small subset of patients progress to testicular infarction, a rare but serious complication. We describe a 73-year-old man with epididymo-orchitis and a pyocele whose initial ultrasound demonstrated reversal of diastolic intratesticular flow, in keeping with impending infarction. Despite transient improvement with antibiotics, he re-presented on day 8 with recurrent pain and near-complete infarction on repeat ultrasound, with persistent reversed diastolic flow in regions of preserved vascularity. This case highlights reversal of diastolic flow as a radiological red flag in epididymo-orchitis and suggests practical recommendations for Doppler assessment, early repeat imaging, and urgent urological review.

## Introduction

Acute scrotal pain is a frequent cause for urological assessment in the Emergency Department (ED), where timely distinction between conditions such as epididymo-orchitis and testicular torsion is critical to avoid irreversible injury. Epididymo-orchitis occurs in approximately 1-2.45 in 1000 men per year and generally responds well to antibiotics.^1^^,^^2^ In a small subset, however—estimated at 3%-4%—infection progresses to testicular infarction, a serious but under-recognized complication.^2^

Predicting infarction remains difficult due to the nonspecific nature of clinical signs such as persistent pain, scrotal edema, and systemic features of infection. Although Doppler ultrasound is the standard imaging modality, its ability to detect early testicular hypoperfusion is limited. Reversal of diastolic flow on spectral Doppler has been described in rare cases as a potential harbinger of infarction.^3–6^

We present the case of a 73-year-old patient who was diagnosed with epididymo-orchitis that progressed to near-complete testicular infarction. The unique contribution of this case is not the first description of reversed diastolic intratesticular flow, but the serial demonstration of progression from a high-resistance Doppler waveform to interval loss of testicular perfusion despite initial symptomatic improvement with antibiotics. The sequence highlights that once reversed diastolic flow is identified, routine outpatient follow-up may be insufficient; instead, urgent urological evaluation, close observation, and early repeat Doppler assessment should be considered.

## Case presentation

A 73-year-old man presented to the ED with a 3 days of increasing right testicular pain associated with gradual scrotal swelling. He had a recent complex hospital admission for ventricular tachycardia arrest requiring emergency transcatheter aortic valve implantation (TAVI) and pacemaker insertion, ICU admission, and a prolonged period of indwelling catheter (IDC) use.

His scrotal symptoms started 3 days after his recent discharge from the hospital. Apart from scrotal pain and swelling, he also reported fever and purulent penile discharge. He denied any irritative or lower urinary symptoms. He had no history of scrotal trauma or recent sexual intercourse.

On physical examination, he was febrile at 38 °C with stable vitals. There was an enlarged, swollen, erythematous right testicle, acutely tender to light palpation. Initial blood tests were notable for a white cell count of 32.7 × 10^9^/L (normal range 4-11 × 10^9^/L) and neutrophil count of 30.4 × 10^9^/L (normal range 2.0-7.5 × 10^9^/L). C-reactive protein (CRP) was elevated at 349 mg/L (normal <5 mg/L). Urinalysis was positive for leukocyte esterase, nitrite, and protein, with >800 leukocytes and 30 erythrocytes per high-power field. A urine culture grew *Pseudomonas aeruginosa*. First-pass urine tests for Chlamydia and gonorrhea were negative.

**Figure 1:**
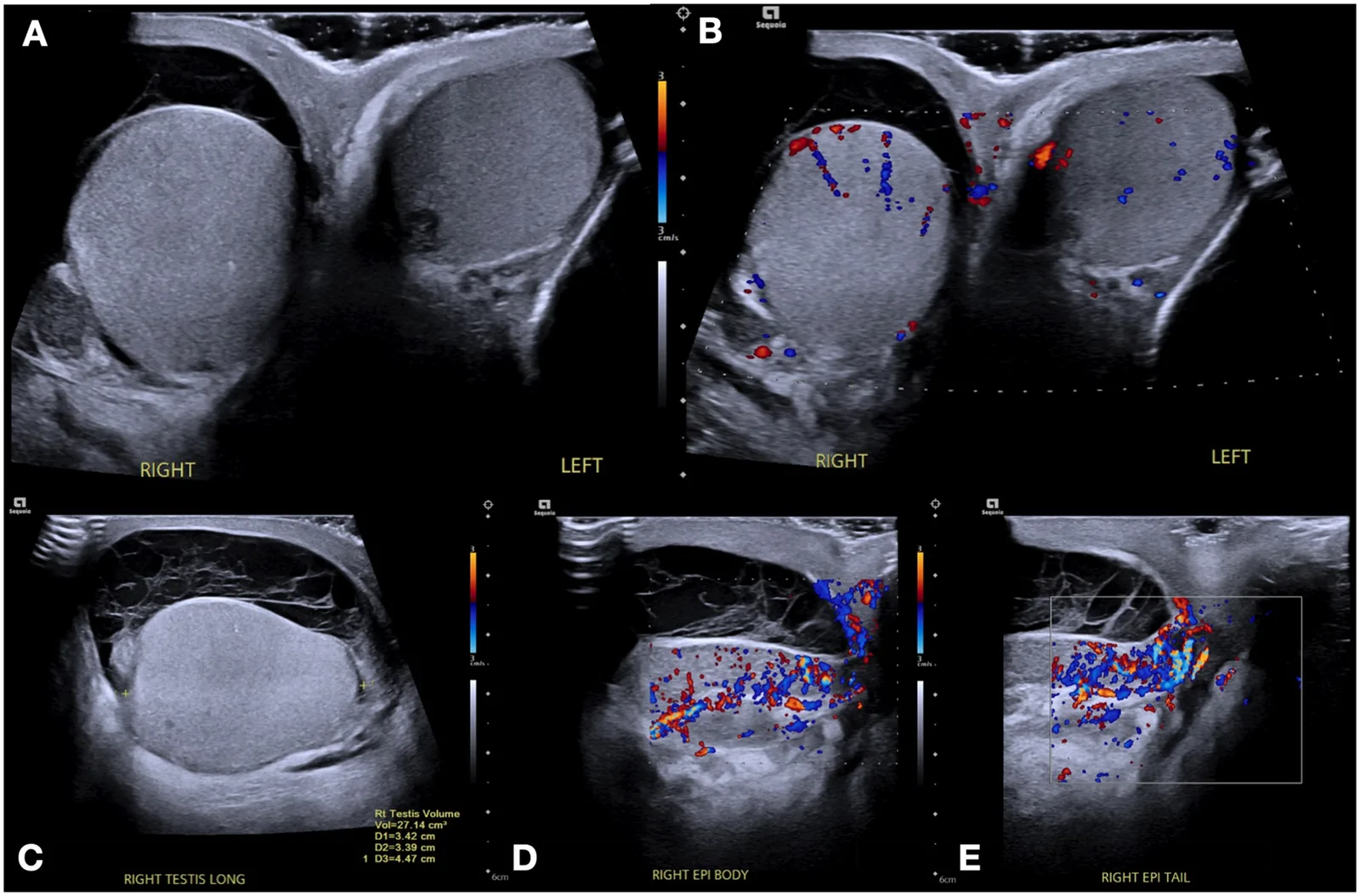
Initial scrotal ultrasound at first ED presentation. Images were obtained using a Siemens ACUSON Sequoia ultrasound machine with an 18L6 linear transducer and testicular preset. Color Doppler images were acquired using a low-flow scale of ±3 cm/s and color PRF of 488 Hz. (A) Transverse gray-scale image of both testes showing an enlarged, edematous right testis with surrounding loculated hydrocele. (B) Transverse color Doppler image demonstrating relatively increased vascular flow in the right testis. (C) Longitudinal gray-scale image showing an enlarged, heterogeneous right testis (34 × 45 × 34 mm) with complex fluid and loculations in the right hemiscrotum, consistent with a pyocele. (D and E) Color Doppler images of the right epididymal body (D) and tail (E) showing edema and hyperemia.

**Figure 2:**
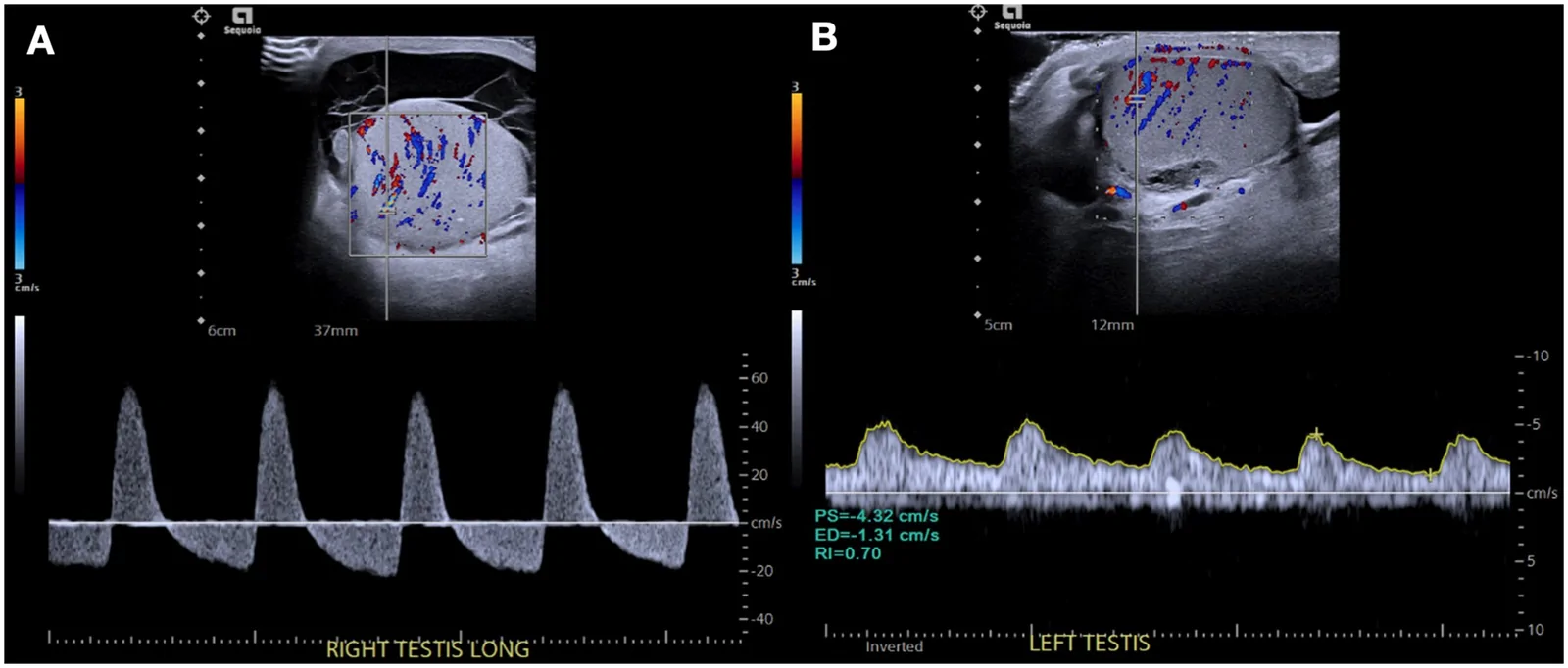
Initial spectral Doppler ultrasound. Images were obtained using a Siemens ACUSON Sequoia ultrasound machine with an 18L6 linear transducer and testicular preset. (A) Pulsed-wave spectral Doppler of the right testis, obtained with a gate size of 1.0, PRF 5208 Hz, and filter setting 40, demonstrating a high-resistance arterial waveform with reversal of diastolic flow and RI >1.0. (B) Pulsed-wave spectral Doppler of an intratesticular artery in the left testis, obtained with a gate size of 1.0, PRF 2000 Hz, and filter setting 40, showing preserved forward diastolic flow with PSV 4.32 cm/s, EDV 1.31 cm/s, and RI 0.70.

On US evaluation, the right testicle and epididymis were thickened and heterogeneous in appearance with increased vascularity in keeping with epididymo-orchitis (Figure 1). Spectral waveform demonstrated reversal of diastolic flow within the intratesticular arteries and a high resistive index (RI > 1.0), concerning for impending infarction (Figure 2A). The contralateral left testis demonstrated a normal arterial waveform for comparison (Figure 2B). A complex loculated hydrocele was also present in the right hemiscrotum, most consistent with a pyocele (Figure 1C).

He was admitted under Urology and commenced on IV ciprofloxacin 400 mg three times daily because of a beta-lactam allergy. General measures, including scrotal elevation and compression were applied. Following initiation of IV antibiotics, there was an interval reduction in scrotal swelling and improvement in pain. He was discharged on day 3 on oral ciprofloxacin 750 mg twice daily for 4 weeks, with planned outpatient review and repeat ultrasound after completing antibiotics.

**Figure 3:**
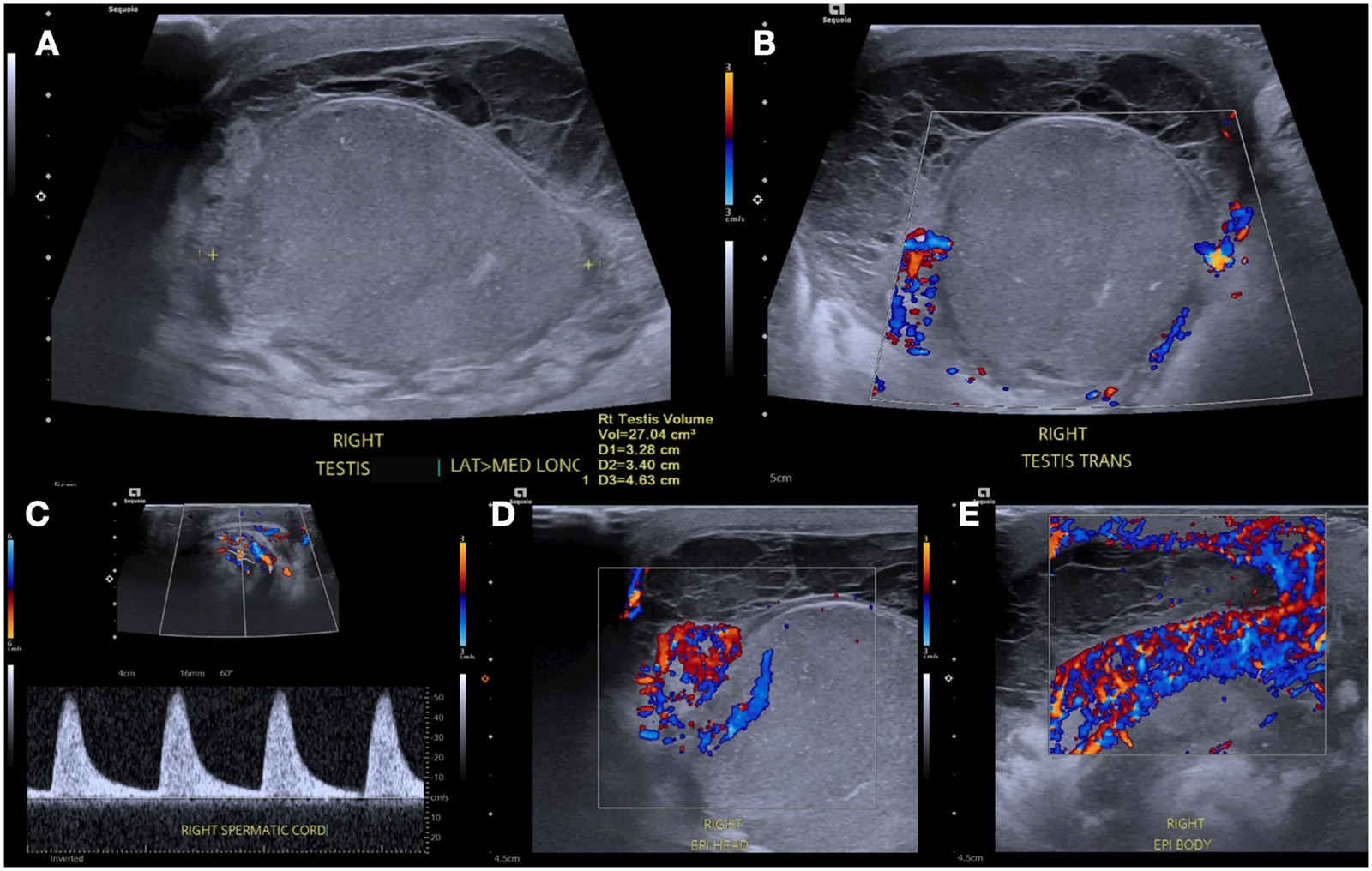
Repeat scrotal ultrasound on day 8. Images were obtained using a Siemens ACUSON Sequoia ultrasound machine with a 15L4 linear transducer and testicular preset. Color Doppler images were acquired using a low-flow scale of ±3 cm/s and color PRF of 625 Hz, except for the spermatic cord spectral Doppler image. (A) Longitudinal gray-scale image showing a persistently enlarged right testis (33 × 46 × 34 mm) with heterogeneous echotexture. (B) Geographic hypoechoic areas with absent Doppler flow, suggestive of interval infarction; a complex loculated hydrocele/pyocele was also present. (C) Pulsed-wave spectral Doppler of the right spermatic cord, obtained with a gate size of 1.5, PRF 4310 Hz, filter setting 60, and Doppler angle 60°, showing preserved arterial waveform. (D and E) Color Doppler images of the right epididymal head (D) and tail (E) showing persistent bulkiness and hyperemia, consistent with ongoing epididymitis.

**Figure 4:**
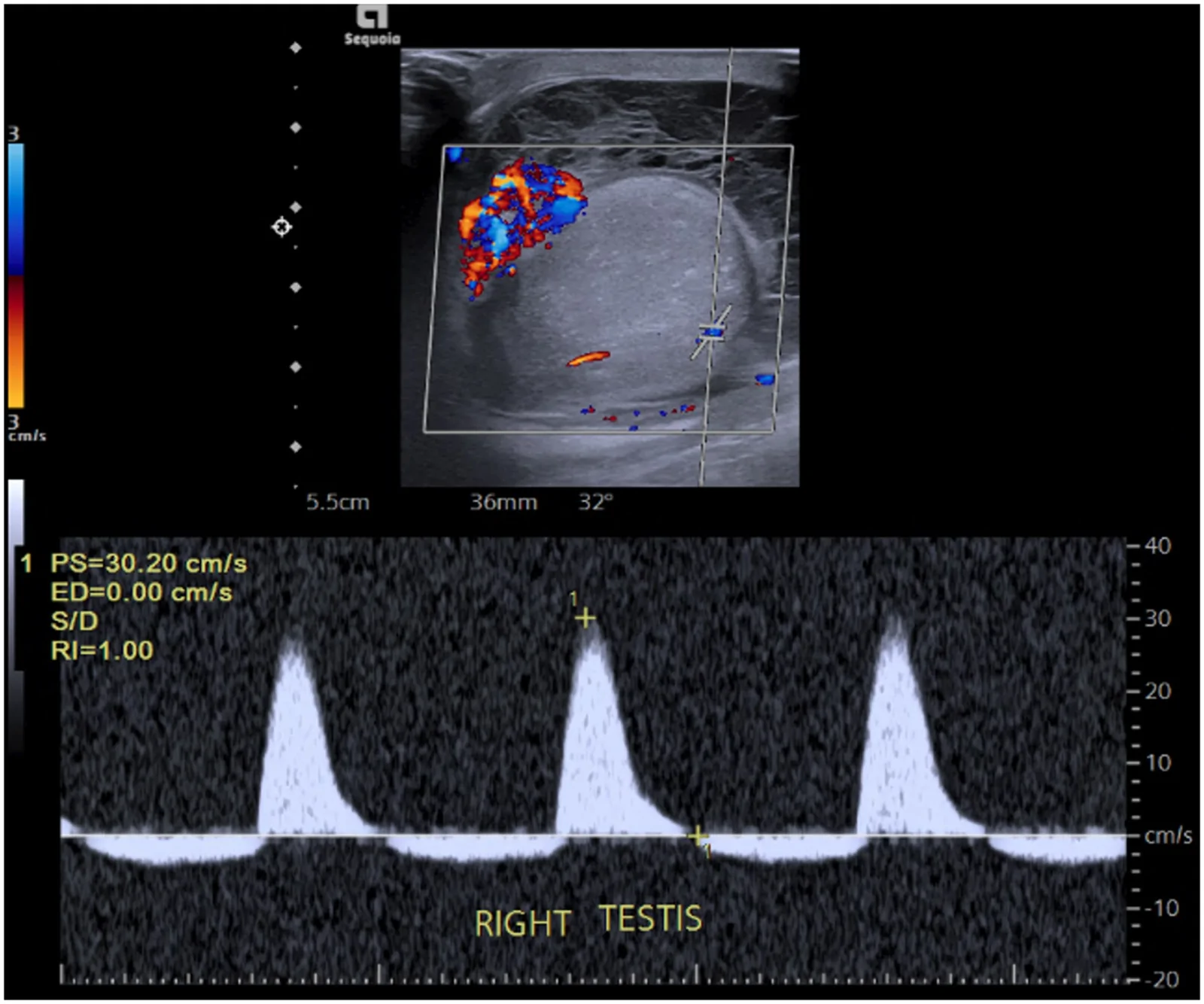
Repeat spectral Doppler ultrasound of the right testis on day 8. Pulsed-wave spectral Doppler of residual intratesticular arterial flow was obtained using a Siemens ACUSON Sequoia ultrasound machine with a 15L4 linear transducer and testicular preset. Color Doppler scale was ±3 cm/s with PRF 625 Hz. Spectral Doppler was obtained with a gate size of 1.5, PRF 5435 Hz, filter setting 60, and Doppler angle 32°, demonstrating persistent high-resistance arterial flow with reversed diastolic flow; PSV 30.20 cm/s, EDV 0.00 cm/s, and RI 1.00.

Unfortunately, he re-presented on day 8 of treatment with an acute worsening of right testicular pain. Despite good compliance with antibiotics, he reported only initial symptomatic improvement during the first 2 days post-discharge. Repeat ultrasound again showed features of right epididymo-orchitis with a complex surrounding hydrocele, however this time, the right testis had large geographic hypoechoic regions with absent internal Doppler flow, consistent with interval infarction (Figure 3). In other areas where residual vascular flow was demonstrated, reversal of diastolic flow was again noted (Figure 4).

The patient was subsequently taken to the theater on day 10. As he was not fit for general anesthesia, scrotal exploration was performed under sedation and local anesthetic. The pyocele was evacuated for symptomatic relief, and the incision was packed with Betadine-soaked ribbon gauze. He was discharged with district nursing support for daily wound care and a further 4-week course of oral ciprofloxacin. Although his symptoms initially improved, scrotal swelling, pain, and discharge recurred shortly after completing antibiotics. Given his comorbidities, he remained unfit for definitive treatment with orchidectomy; ongoing management was therefore limited to symptomatic measures with repeated antibiotics and local wound care.

## Discussion

Testicular ischemia resulting from epididymitis is an infrequently recognized and often belatedly diagnosed complication, which significantly limits opportunities for testicular salvage. While testicular atrophy following epididymitis is a known sequela, the true incidence of testicular necrosis may be underreported. This underestimation likely arises because patients with persistent symptoms and necrotic testicular tissue found on exploration are sometimes misdiagnosed with testicular torsion, obscuring the role of severe epididymitis in causing infarction.^7^

The pathophysiology centers on vascular compromise due to the close anatomical relationship between the testicular vessels and the inflamed epididymis. Initial compression of testicular arteries, veins, and lymphatics results from swelling of the epididymis, followed by further constriction due to funiculitis affecting the spermatic cord.^8^ Initially, venous and lymphatic outflow is impaired by edema within the confined space of the spermatic fascia. As pressure escalates, arterial perfusion may become compromised due to compression or secondary thrombosis, with bacterial endotoxins further exacerbating vascular injury.^8^^,^^9^ This cascade can culminate in irreversible ischemic damage. Clinically, scrotal edema, severe pain, and poor response to antibiotics should raise suspicion for developing ischemia.^9^

Color and spectral Doppler ultrasonography provide complementary information in epididymo-orchitis. Color Doppler demonstrates the presence and distribution of blood flow and typically shows hyperemia, whereas spectral Doppler assesses arterial waveforms and vascular resistance. As a result, color Doppler alone may miss early hemodynamic changes that precede infarction. Spectral Doppler should therefore be included in scrotal ultrasound assessment to identify high-resistance flow patterns. Reversal of diastolic arterial flow is a distinctive high-resistance waveform that reflects markedly increased downstream vascular resistance and impaired diastolic perfusion of the testicular parenchyma, signaling impending testicular infarction.^3–6^

Multiple case reports have supported the clinical importance of this finding. Sanders et al. described a patient with epididymo-orchitis who exhibited reversed diastolic flow and was found to have a nonviable testicle during surgical exploration. Histopathology confirmed suppurative epididymitis with ischemic necrosis.^3^ Similarly, Gerscovich et al. reported delayed testicular infarction in a patient managed conservatively despite the presence of reversed diastolic flow. Subsequent imaging revealed absent intratesticular flow and scrotal exploration confirmed gangrenous testicular tissue.^4^ Rhudd et al. highlighted the risk of delayed infarction even after an initially negative surgical exploration. Although no ischemia was found during the procedure, absent flow was observed the next day and orchiectomy was ultimately required.^5^ Marks et al. described a patient whose initial Doppler findings were normal. However, reversed diastolic flow later developed and was followed by loss of arterial flow and confirmed infarction.^6^ In contrast, Wang et al. reported a case where reversed diastolic flow resolved after urgent scrotal exploration and conservative management. The patient recovered without testicular loss and follow-up imaging showed no further evidence of ischemia.^10^

A focused appraisal of these 5 published cases reveals 3 consistent themes.^3–6^^,^^10^ First, reversal of diastolic arterial flow on testicular Doppler ultrasound preceded the complete loss of testicular perfusion. Second, 4 testes were lost when exploration was delayed and antibiotics discontinued after roughly 2 weeks, whereas the single salvaged testis underwent same-day decompressive exploration, early repeat ultrasound within 24 h, and a 5-week course of broad-spectrum antibiotics. Third, *Escherichia coli* and *Pseudomonas aeruginosa* were the pathogens most often isolated. This microbial pattern is echoed by Fehily et al., who reviewed 10 cases of infarction after bacterial epididymo-orchitis and likewise found *E. coli* and *P. aeruginosa* to be the commonest organisms, suggesting that certain pathogens may have a greater propensity to contribute to vascular compromise.^9^ This observation raises the possibility that the type of infective organism may also influence the risk of progression to infarction, either through differences in virulence, toxin production, or inflammatory response. Patients in these reports were typically older, catheter dependent or recently hospitalized; metabolic disease and immunosuppression were additional risk factors. Given the limited number of published cases, these observations should be interpreted cautiously and regarded as suggestive rather than definitive.

In this limited case-based context, these observations suggest the following practical considerations. First, an intratesticular arterial waveform should be obtained on every scrotal ultrasound so that early reversal is not missed. Second, isolation of *E. coli* or *P. aeruginosa* in an elderly patient who is catheter dependent or carries additional comorbid risk factors should prompt closer monitoring and early repeat imaging. Third, once reversal of diastolic flow is identified, early urology review is highly recommended. Exploration should be considered because it offers the greatest likelihood of salvage, but where nonoperative management is chosen, an extended intravenous-led antibiotic course (typically 4-6 weeks) with repeat Doppler within 24 h to reassess perfusion is advised.

In interpreting the repeat ultrasound, intratesticular abscess was an important differential diagnosis, particularly in the setting of epididymo-orchitis and an associated pyocele. However, infarction was favored because the initial high-resistance waveform with reversed diastolic flow progressed to geographic hypoechoic regions with absent internal Doppler flow, rather than a discrete complex intratesticular collection. Although histological confirmation was not available because the patient was unfit for orchidectomy, and long-term imaging follow-up was limited, the serial clinical and Doppler evolution is most consistent with progressive testicular infarction. This supports treating reversed diastolic intratesticular arterial flow as a radiological red flag that warrants urgent escalation and early reassessment rather than routine outpatient follow-up.

## Conclusion

In conclusion, this case highlights the need to integrate clinical suspicion with radiological evidence in patients with epididymo-orchitis. Recognition of abnormal Doppler waveforms, especially reversal of diastolic flow, in the context of epididymo-orchitis should prompt urgent clinical reassessment. While this finding is not invariably associated with infarction, the high frequency of testicular loss in documented cases warrants urgent Urological review with consideration of early surgical exploration. Improved identification of patients at risk through detailed Doppler assessment and understanding of waveform evaluation may allow timely intervention and improved testicular salvage rates.

## Learning points

- Reversed diastolic intratesticular arterial flow on spectral Doppler in epididymo-orchitis is a red flag for impending infarction.
- Clinical improvement on antibiotics does not exclude progression to infarction.
- Scrotal ultrasound for suspected epididymo-orchitis should include intratesticular arterial waveforms (not color Doppler alone).
- If reversal is detected, urgent urology review and a low threshold for exploration should be considered.
- If managed nonoperatively, close monitoring with repeat Doppler (within 24 h) and prolonged antibiotics (often 4-6 weeks) should be considered.
